# Supplementary material for: Therapeutic Fasting: Are Patients Aged 65 and Over Ready?
Source: Nutrients. 2022 May 10;14(10):2001. doi: 10.3390/nu14102001 (PMC9143805; doi:10.3390/nu14102001)
Supplement: Supplementary file 1 [file nutrients-14-02001-s001.zip › nutrients-1692305-supplementary.pdf]

FASTING, AND YOU ?

Are you over 65 years old? We are interested in your opinion!

Here is an individual and anonymous questionnaire about therapeutic fasting which should take you less than 5 minutes to complete. This survey was created with the sole purpose of collecting your personal opinion on this subject, so there is no right or wrong answer.

For your information, therapeutic fasting consists of a voluntary partial or complete deprivation of food, without reduction of water consumption, for a few days, with the expected objective of improving health.

PART 1: YOUR OPINION ON THERAPEUTIC FASTING

- **Your perception of therapeutic fasting: (indicate with a cross your level of approval to the 4 propositions below)**

	Totally agree	Agree	Neither agree nor disagree	Disagree	Totally disagree
For you, therapeutic fasting :					
1) Is an interesting topic					
2) May be useful					
3) May be dangerous					
4) Scares you					

- **Before this questionnaire, have you ever heard of therapeutic fasting?**
  ☐ Yes, a lot ☐ Yes, a little ☐ I do not know ☐ No

- **Have you ever practiced fasting?**
  ☐ Yes ☐ No ☐ I do not remember

- **If yes, for what reason?**
  ☐ Religion (Lent, Ramadan,...)
  ☐ Health (therapeutic fasting)
  ☐ Sport
  ☐ Voluntary weight loss
  ☐ Discovery
  ☐ Other (precise) : ………..

- **If yes, you have found it:**

Very hard	Hard	Neither easy nor hard	Easy	Very easy

- **The questions below refer to a 3-day therapeutic fast, which would be performed under medical supervision, during which food intake would be reduced by three-quarters (e.g., one broth per meal with one bowl of rice per day) without reducing water intake:**

	Yes, a lot	Yes	I do not know	No, rather not	No, not at all
Do you think you have the physical ability to perform this therapeutic fasting?					
Do you think you have the mental resources to perform this therapeutic fasting?					
Do you think this therapeutic fasting is compatible with your lifestyle?					
Would you be willing to adapt your daily activities to be able to perform this therapeutic fasting?					
Do you think that if you were younger than 65, your attitude or outlook on fasting would have been different?					
Would you feel comfortable telling your primary care physician that you are doing this?					
In general, do you think people have a positive view about therapeutic fasting?					
Would you be willing to perform this therapeutic fasting if you were told that it:					
1) improves your overall health?					
2) decreases the burden of chronic diseases (such as high blood pressure, diabetes, etc.)?					
3) reduces the number of medications you have to take every day?					
4) reduces the side effects of specific heavy treatments (chemotherapy for example)					
5) improves your immune defenses against infections?					
6) improves the effectiveness of vaccines?					

- If you were offered a therapeutic fasting program, what are some of the ways that would facilitate your participation and adherence to the program:

	Totally agree	Agree	Neither agree nor disagree	Disagree	Totally disagree
The practice of a short fast of less than 4 days.					
The annual repetition of this program.					
A favorable opinion from your attending primary care physician.					
Fasting with your friends and/or family.					
Having a medical follow-up during the fasting period.					

PART 2: YOUR SITUATION

- **Are you :**
  - ☐ A woman
  - ☐ A man

- **What is your year of birth?** |__|__|__|__|

- **Currently, you are living:**
  - ☐ Alone
  - ☐ In couple
  - ☐ With another family member
  - ☐ Other:……………………………

- **Currently, you reside in a city or town with:**
  - ☐ Less than 1000 inhabitants
  - ☐ Between 1000 and 10000 inhabitants
  - ☐ More than 10000 inhabitants
  - ☐ I do not know

- **Your level of education is:**
  - ☐ No diploma
  - ☐ Elementary school
  - ☐ Junior high school
  - ☐ High school
  - ☐ Undergraduate and graduate

- **Over the past 6 months, how would you rate your health status compared to people of the same age?**

Really healthy	Healthier	Not better nor worse	In poorer health	Really unhealthy
